# Deep Evolutionary Conservation of an Intramolecular Protein Kinase Activation Mechanism

**DOI:** 10.1371/journal.pone.0029702

**Published:** 2012-01-03

**Authors:** Jingfen Han, Diego Miranda-Saavedra, Nathan Luebbering, Aman Singh, Gary Sibbet, Michael A. J. Ferguson, Vaughn Cleghon

**Affiliations:** 1 Division of Developmental Biology, Cincinnati Children's Hospital Medical Center, Cincinnati, Ohio, United States of America; 2 WPI Immunology Frontier Research Center (IFReC) Osaka University, Osaka, Japan; 3 Integrated Program in Biomedical Sciences, University of Tennessee Health Science Center, Memphis, Tennessee, United States of America; 4 Beatson Institute for Cancer Research, Garscube Estate, Glasgow, United Kingdom; 5 Division of Biological Chemistry and Drug Discovery, College of Life Sciences, University of Dundee, Dundee, United Kingdom; Institute of Molecular and Cell Biology, Singapore

## Abstract

DYRK-family kinases employ an intramolecular mechanism to autophosphorylate a critical tyrosine residue in the activation loop. Once phosphorylated, DYRKs lose tyrosine kinase activity and function as serine/threonine kinases. DYRKs have been characterized in organisms from yeast to human; however, all entities belong to the Unikont supergroup, only one of five eukaryotic supergroups. To assess the evolutionary age and conservation of the DYRK intramolecular kinase-activation mechanism, we surveyed 21 genomes representing four of the five eukaryotic supergroups for the presence of DYRKs. We also analyzed the activation mechanism of the sole DYRK (class 2 DYRK) present in *Trypanosoma brucei* (TbDYRK2), a member of the excavate supergroup and separated from Drosophila by ∼850 million years. Bioinformatics showed the DYRKs clustering into five known subfamilies, class 1, class 2, Yaks, HIPKs and Prp4s. Only class 2 DYRKs were present in all four supergroups. These diverse class 2 DYRKs also exhibited conservation of N-terminal NAPA regions located outside of the kinase domain, and were shown to have an essential role in activation loop autophosphorylation of *Drosophila* DmDYRK2. Class 2 TbDYRK2 required the activation loop tyrosine conserved in other DYRKs, the NAPA regions were critical for this autophosphorylation event, and the NAPA-regions of *Trypanosoma* and human DYRK2 complemented autophosphorylation by the kinase domain of DmDYRK2 in trans. Finally, sequential deletion analysis was used to further define the minimal region required for trans-complementation. Our analysis provides strong evidence that class 2 DYRKs were present in the primordial or root eukaryote, and suggest this subgroup may be the oldest, founding member of the DYRK family. The conservation of activation loop autophosphorylation demonstrates that kinase self-activation mechanisms are also primitive.

## Introduction

Phosphorylation of activation loop residues is a wide-spread and important mechanism for regulating the catalytic activity of a protein kinase. Activation loop phosphorylation may be carried out by a separate protein kinase as part of a signaling cascade, or a kinase can autophosphorylate using an intermolecular or intramolecular mechanism [1]–[2]. DYRKs (dual-specificity tyrosine phosphorylation-regulated kinases) and glycogen synthase kinase 3s (GSK3s) are dual-specificity kinases that autophosphorylate a critical tyrosine residue in the activation loop region of the kinase domain during maturation of the molecule [3]–[4]. Once the residue is phosphorylated, DYRKs and GSK3s lose their tyrosine kinase activity and function only as serine/threonine kinases [3]–[12]. These dual-specificity kinases face the dilemma of temporarily converting what is essentially a serine/threonine kinase into a tyrosine kinase. For GSK3, we demonstrated that the chaperone Hsp90 was necessary for the tyrosine autophosphorylation event but was not needed for the subsequent phosphorylation of substrates on serine/threonine [4]. Recently, non-catalytic regions in the C-terminus of GSK-3 were also shown to be essential for intramolecular tyrosine phosphorylation [13].

The DYRK family can be further subdivided into five subfamilies: class 1 (or DYRK1), class 2 (or DYRK2), Yaks, HIPKs (homeodomain-interacting protein kinase), and PRP4s (pre-messenger RNA [mRNA]-processing protein 4) [14]–[15]. Increasingly, DYRK members are implicated in a variety of developmental and disease processes. The founding member of the DYRKs, Yak1p, regulates nutrient starvation or oxidative stress-induced cell cycle arrest in *Saccharomyces cerevisiae* by acting as a functional antagonist to the Ras/PKA and target of rapamycin (TOR) pathways [16]–[17]; and in *Dictyostelium*, the homologous Yak1p regulates a starvation-induced growth arrest initiating a transition from vegetative growth to a developmental phase [18]. Class 1 DYRK orthologues, minibrain and DYRK1A, are essential for post embryonic neurogenesis of the optic lobes and central brain hemispheres in *Drosophila* and mice, respectively [19]–[21]. The closely related mouse class 1 paralogue, DYRK1B, is involved in myoblast cell differentiation [22]. Class 2 DYRK Pom1p from *S. pombe* provides positional information for both cell growth and cell division [23] via regulation of the G_2_-M transition [24]–[25]. In *Caenorhabditis elegans*, class 2 DYRK, MBK-2, promotes the oocyte-to embryo transition, and modulates asymmetrical cell division and the patterning of the embryonic axis [26]–[28]. Human DYRK2 is a critical component in the Hedgehog signaling pathway [29]; acts as an molecular assembler of an E3 ubiquitin ligase complex, which plays a crucial role in regulating normal mitotic progression [30]; and functions in the DNA damage signaling pathway by phosphorylating p53 thereby initiating a p53 apoptotic response [31]. The mammalian paralogue, DYRK3, is involved in erythropoiesis in mammals [32].

Class 1 and 2 DYRKs are the best-characterized subfamilies, particularly with regard to understanding activation mechanisms or regulatory events controlling the kinase activity of these enzymes. Class 1 and 2 subfamily members share a conserved central kinase domain and an adjacent N-terminal DH-box (DYRK homology box) but have divergent extended N- and C-terminal regions [33]. A variety of mechanisms exist to control DYRK catalytic activity. Mammalian DYRK4 is alternatively spliced to regulate the intracellular localization and catalytic activity of the protein [12]. In yeast *S. cerevisiae*, Yak1p is translocated to the nucleus in response to TOR signaling or glucose depletion and thereby gains access to relevant substrates [24], [34]–[35]. Similarly, in response to DNA damage mammalian DYRK2 translocates to the nucleus and phosphorylates p53 thereby initiating an apoptotic response [31]. The activity of the *C. elegans* DYRK family member, MBK-2, is carefully regulated during the oocyte-to-embryo transition through a series of actions involving timely relocalization of the kinase thus providing access to relevant substrates [36], inhibitory protein complexes involving the pseudophosphatases EGG-3, EGG-4 and EGG-5 [36]–[38], and finally through direct phosphorylation of MBK-2 by the cell cycle kinase, CDK-1 [37]. However, the best understood mechanism for regulating the catalytic activity of the kinase is phosphorylation of the DYRK activation loop.

All class 1 and 2 DYRKs as well as YAKs characterized to date contain a YxY motif in the activation loop; but only the second tyrosine residue in this motif is phosphorylated, an autocatalytic event that is essential for full serine/threonine kinase activity of the protein [3], [6], [9], [12]. The phosphorylation of the activation loop tyrosine is an intramolecular event, mediated by a short-lived transitional intermediate [3]. During maturation of the DYRK protein, the transitional intermediate adopts a conformational change that enables intramolecular phosphorylation of the consensus activation loop tyrosine. Recently we identified regions in the non-catalytic N-terminus (N-terminal autophosphorylation accessory or NAPA regions) of the *Drosophila* class 2 DYRK, DmDYRK2 (also known as smi35A). Although the NAPA region was located outside of the kinase domain, we demonstrated that it was essential for activation loop phosphorylation but not for the subsequent serine/threonine phosphorylation of substrates [39]. DYRKs are essentially serine/threonine kinases. We postulated that the role of the NAPA domain was to transiently convert the DYRK protein into an intramolecular tyrosine kinase for activation loop phosphorylation.

Eukaryotes derive from a common ancestor. The advent of modern molecular biology including DNA sequencing has provided new insights into the relatedness and evolution of eukaryotic organisms leading to a re-examination of the eukaryotic phylogenetic tree. Based on accumulating evidence from morphological comparisons, biochemical pathways, and molecular phylogenetic approaches current evolutionary hypotheses have assembled eukaryotes into five or six supergroups [40]–[41]. Currently DYRK family members have been studied in yeast, *Dictyostelium*, *C. elegans*, *Drosophila*, and mammals (see references [14] and [15] for review). Although diverse, all of these life forms come from a single supergroup, the unikonts.

In this report, we have taken advantage of the availability of completely sequenced genomes from highly diverse organisms to interrogate the evolutionary conservation and phylogenetic distribution of DYRK family members. We have also compared NAPA region function across evolutionary time, by cloning and analyzing the sole class 2 DYRK encoded by T. *brucei* (TbDYRK2). Here we show that, similar to other DYRKs, TbDYRK2 is phosphorylated on the conserved activation loop tyrosine, and phosphorylation of this residue is necessary for full serine/threonine kinase activity. In addition, deletion of the NAPA-1 region or point mutations within the NAPA-2 region eliminated tyrosine autophosphorylation. Finally, we demonstrate that the isolated NAPA-regions of TbDYRK2 and human DYRK2 were able to complement tyrosine autophosphorylation of *Drosophila* DmDYRK2 in *trans* providing evidence for the functional conservation of the NAPA-domain across species.

Our analysis demonstrates the evolutionary ancient nature of the DYRK family, and provides evidence that class 2 DYRKs were present in the primordial or root eukaryote. Our functional analysis of TbDYRK2 provides further evidence for the conservation of the mechanism of intramolecular phosphorylation of the DYRK activation loop and the potential role of the NAPA domain in this process.

## Materials and Methods

### Chemicals and reagents

Anti-Flag M2-agarose and anti-Flag antibodies were purchased from Sigma (St. Louis, MO). Anti-phosphotyrosine 4G10, was purchased from Millipore (Temecula, CA). Horseradish peroxidase conjugated with anti-mouse IgG, protein G sepharase, were obtained from GE Healthcare (Amersham, UK). The QuickChange site-directed mutagenesis kit was from Stratagene (La Jolla, CA). Clean-blot IP detection reagent (HRP) was purchased from Thermo Scientific (Logan, Utah). The SuperSignal west pico chemiluminescent reagent for immunoblot detection was from Amersham Biosciences Inc. (Piscataway, NJ). All PCR and mutagenesis primers were synthesized by Integrated DNA Technologies (Skokie, IL).

### Baculovirus constructs

The open reading frame from the DYRK2 gene of *T. brucei* was amplified from genomic DNA by PCR. The nucleotide sequence and conceptual translation are shown (Fig. S1). At the 5′-end of the coding sequence, we inserted an EcoR1 restriction site, consensus Kozak sequences, an initiating-Met ATG codon, and FLAG-epitope in frame with the protein coding sequence. At the 3′-end of the gene, a second EcoR1 site was added (Fig. S1). The EcoR1-EcoR1 fragment was then cloned into EcoR1 linearized pcDNA3 to generate WT-TbDYRK2. Constructs encoding mutant forms of the protein (K138M-, Y269F-, ΔNAPA1-, ΔN2-, and D103V-TbDYRK2) were generated with the QuikChange site-directed mutagenesis kit (Stratagene) using appropriate mutagenesis primers and FLAG-tagged WT-TbDYRK2 as template. For the Nt-TbDYRK2 mutant, we replaced the single FLAG epitope with a 3X FLAG epitope and added three tandem in-frame stop codons following TbDYRK2 sequences encoding amino acids 1–109 using WT-TbDYRK2 as template.

The generation of baculoviruses expressing FLAG epitope-tagged WT-, K227M-, ΔN1-, and ΔN2-DmDYRK2 has been described previously [39]. To clone additional DmDYRK2 deletion mutants, an initial template construct (Nt-DmDYRK2) consisting of: a 5′ EcoR1 restriction site; initiating ATG and in-frame 3X FLAG epitope followed by DmDYRK2 sequences encoding amino acids 1–197 of the protein followed by three tandem in-frame stop codons; and a 5′ Not1 restriction site was created by PCR from full length DmDYRK2 in pcDNA3. The PCR product was inserted into EcoRI – NotI digested pcDNA3. Nt-NAPA2-DmDYRK2 and Nt-DH-NAPA2-DmDYRK2 were then created by the introduction of three tandem in-frame stop codons at the appropriate location. Nt-ΔDH-DmDYRK2 and DmD2-Nt-Δ(1–120) were generated with the QuikChange site-directed mutagenesis kit using Nt-DmDYRK2 in pcDNA3 as template.

Human Nt-DYRK2 was constructed using the first 447 nucleotides of the Dyrk2 isoform 1 (NM_003583) open reading frame (encoding the first 149 amino acids of the protein). To the 5′-end of the coding sequences, we added an EcoR1 restriction site and consensus Kozak sequences; and in frame FLAG-epitope encoding sequence immediately following the initiating-Met ATG codon. At the 3′-end of the coding sequences, a stop codon followed by a second EcoR1 site was added. The EcoR1-EcoR1 fragment was then cloned into EcoR1 linearized pcDNA3 to generate HsNt-DYRK2.

Constructs encoding the various proteins were then subcloned into the baculoviral shuttle vector pVL1393 (BD Biosciences). The various pVL1393 constructs were then used in conjunction with BaculoGold DNA (BD Biosciences) to generate recombinant baculovirus for expression in Sf9 (Spodoptera frugiperda 9) cells as described previously [39].

### Immunoprecipitation and Western blotting

For most immunoprecipitations, Sf9 cell extracts (0.15 mg) were incubated with Protein G-Sepharose conjugated to anti-FLAG (Sigma) or anti-DmDYRK2 antibody for 1 h on a rotating platform at 4°C. Efficient immunoprecipitation of ΔN2-TbDYRK2 required longer incubation times with the anti-FLAG antibody (up to 10 h). Immunoprecipitates were pelleted, washed twice with 1 ml buffer A [20 mM tris-HCl (pH 8.0), 137 mM NaCl, 10% glycerol, and 1% Igepal CA-630] and once with 1 ml of ice-cold TBS buffer (20 mM tris-HCl [pH 7.6], 137 mM NaCl). Immunoprecipitated proteins were separated by SDS–polyacrylamide gradient gel (4–20% NuPAGE gels) electrophoresis (SDS-PAGE). Proteins were transferred to nitrocellulose membranes, blocked with 3% bovine serum albumin in 20 mM tris-HCl (pH 7.6), 137 mM NaCl, containing 0.2% Tween-20 (TBS-T), and probed with a monoclonal anti-FLAG antibody (1∶5000 dilution) or anti-phosphotyrosine antibody (1∶2000 dilution). Immunoblots were then incubated with HRP horseradish peroxidase conjugate (1∶1000) dilution, and visualized by SuperSignal west pico chemiluminescent reagent detection according to the protocol of the manufacturer (Amersham Biosciences).

### Kinase assays

For peptide kinase assays, WT and mutant forms of TbDYRK2 were immunoprecipitated in triplicate, washed three times in lysis (1% NP40 buffer) buffer, washed once in kinase buffer (30 mM HEPES [pH 7.4], 10 mM MgCl_2_, 1 mM DTT), and incubated with kinase buffer containing ATP and 50 microM Woodtide as described previously [39].

### Identification and classification of DYRKs

The identification of DYRKs was done by scanning the predicted peptide datasets from 21 distinct eukaryotic genomes (Table 1) with a multi-level profile hidden Markov model (HMM) library of the protein kinase superfamily [42], and as detailed in Martin et al [43]. Homology relationships to the characterized DYRKs of model organisms (human, mouse, fly, worm, and the slime mold) were identified by building and curating multiple sequence alignments of protein kinases of the CMGC group. Alignments were built and curated using Jalview [44] and the Neighbor-Joining trees with TOPALi v2 [45]. The bootstrap values reported are based on 1000 replicates.

**Table 1:** Classification and statistics of the 21 eukaryotic genomes scanned for DYRKs.

Taxonomy and species	Common name/description	Source database (version)	Predicted number of protein genes	Number of DYRKs
**Unikonts**				
*Ciona intestinalis*	*Sea squirt*	JGI (2.0)	14002	3
*Entamoeba histolytica*	*Protist parasite*	Kinomer	9766	2
*Gallus gallus*	*Chicken*	Kinomer	22194	7
*Monosiga brevicollis*	*Choanoflagellate*	JGI (1.0)	9196	4
*Nematostella vectensis*	*Sea anemone*	JGI (1.0)	27273	5
*Xenopus tropicalis*	*Western clawed frog (diploid)*	JGI (4.1)	27710	6
**Chromalveolates**				
*Phaeodactylum tricornutum*	*Marine diatom, photosynthetic*	*JGI (2.0)*	*10402*	*3*
*Phytophthora ramorum*	*Water molds*	*JGI (1.1)*	*15743*	*3*
*Phytophthora sojae*		*JGI (1.1)*	*19027*	*2*
*Thalassiosira pseudonana*	*Marine diatom, phytoplankton*	*JGI (3.0)*	*11390*	*3*
**Excavates**				
*Leishmania major*	*Causes Leishmaniasis*	*TriTrypDB (2.2)*	*8408*	*1*
*Trypanosoma brucei*	*Causes Sleeping sickness*	*TriTrypDB (2.2)*	*9895*	*1*
*Trypanosoma cruzi*	*Causes Chagas' disease*	*TriTrypDB (2.2)*	*10813*	*1*
**Plants**				
*Arabidopsis thaliana*	*Model plant*	*Kinomer*	*30690*	*4*
*Chlamydomonas reinhardtii*	*Unicellular alga, photosynthetic*	*JGI (4.0)*	*15256*	*2*
*Cyanidioschyzon merolae*	*Primitive red alga living in acidic hot springs*	*U Tokyo (April 13, 2004)*	*5331*	*1*
*Ostreococcus lucimarinus*	*Early-diverging unicellular green algae*	*Kinomer*	*7651*	*2*
*Ostreococcus tauri*		*Kinomer*	*7892*	*2*
*Physcomitrella patens*	*First sequenced moss, basal to land plants*	*JGI (1.1)*	*35938*	*8*
*Populus trichocarpa*	*Poplar tree*	*JGI (1.1)*	*45555*	*4*
*Volvox carteri*	*Multicellular chlorophyte alga*	*JGI (Assembly V2)*	*15544*	*2*

## Results

### Class 2 DYRKs, the oldest subgroup of the DYRK family

**Figure 1:**
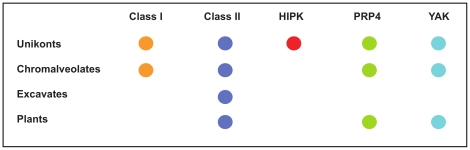
The evolutionary age of the DYRK subfamilies. DYRK family members identified in our search of 21 sequenced genomes clustered into five separate subfamilies (class 1, class 2, HIPK, PRP4, and Yak). Individual DYRK subfamilies were then placed into their respective eukaryotic supergroup.

Recently, genome sequencing projects have made available information that allows us to ask questions concerning the conservation and evolution of proteins and protein motifs. We were interested in comparing the evolutionary depth of the various DYRK subfamilies. Keeling et al [41] suggest that eukaryotes are constituted by five major ‘supergroups’ (Chromalveolates, Excavates, Plantae, Rhizaria and Unikonts). Complete genome sequence data is available for organisms from all supergroups except the Rhizaria. We performed searches for DYRKs in 21 completely sequenced genomes using a sophisticated multi-level profile hidden Markov model (HMM) library of the protein kinase superfamily [42]. DYRK family members are found in all supergroups surveyed (Table 1 and Table S1). The phylogenetic tree resulting from our analysis shows that the DYRK-related proteins cluster into five distinct subfamilies: Class 1 and 2 DYRKs, HIPKs, Yaks, and Prp4s (Fig. S2 and Table S1). Interestingly, HIPKs appear to be present only in Unikonts, and thus are likely to represent a late innovation. Class 1 DYRKs were found in two supergroups, Chromalveolates and Unikonts; whereas Yaks and Prp4s were present in three of the four supergroups (absent from Exchavates). Strikingly, only Class 2 DYRKs were found in all four supergroups (Fig. 1, Fig. S2 and Table S1).

As this broad representation of class 2 DYRKs has not been previously compared, we performed a multiple protein alignment using our class 2 dataset. The NAPA regions as well as the DH-box were found to be conserved in the class 2 DYRKs identified in all four eukaryotic supergroups (Fig. S3). These results suggest that Class 2 DYRKs are the founding members of the family, and that the NAPA domain is intrinsic to this DYRK subfamily.

### Expression and phosphorylation of recombinant TbDYRK2

**Figure 2:**
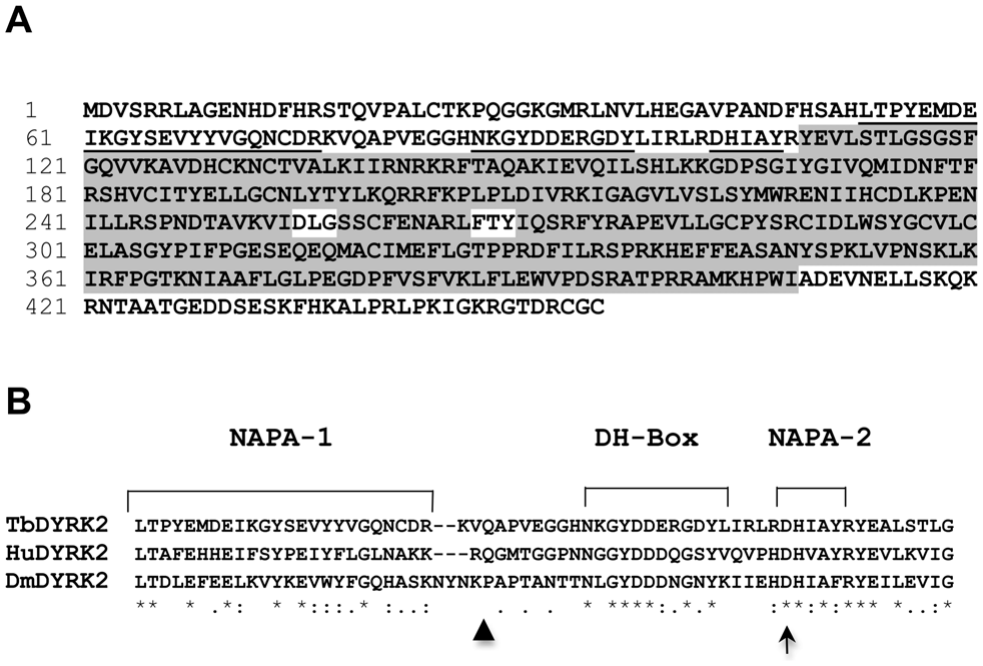
Class 2 DYRK from *Trypanosoma brucei*. (A) Amino acid sequence of TbDYRK2. The conserved NAPA-1, DH-box, and NAPA-2 regions are underlined; the central kinase domain is boxed in gray; and the atypical activation loop sequences D-L-G and F-T-Y are boxed in white. (B) Multiple protein alignment of the NAPA and DH-box regions of TbDYRK2, DmDYRK2 and HsDYRK2. Symbols * invariant, : conservative substitutions, and . semi-conserved substitutions. TbDYRK2 mutant proteins affecting the NAPA regions as described later include ΔNAPA-1 (removal of only the indicated NAPA-1 region), ΔN2 (all N-terminal residues up to lower triangle removed), and D103V (conserved Asp in NAPA-2 altered to Val indicated by lower arrow).

To functionally compare class 2 DYRKs across evolutionary time, we cloned, and analyzed the sole DYRK family member, a class 2 DYRK, identified in the excavate *Trypanosoma brucei*. The sequence of the gene from *T. brucei* strain 427 (Fig. S1) and 455 amino acid gene-product (Fig. 2A) are shown. The sequences of the homologous genes from *T. brucei* strains TREU927 and DAL972 have also been reported and the three proteins are almost identical (three amino acids show heterogeneity). TbDYRK2 is one of the shortest of the class 2 DYRKs containing 108 N-terminal amino acids, a central kinase domain of 300 residues, and a C-terminus of 47 amino acids (Fig. 2A). The protein contains all the canonical features of a class 2 DYRK including the conserved NAPA-1, NAPA-2, and DH-box regions (Fig. 2B). However, TbDYRK2 has several notable features including amino acids FTY in the activation loop of the kinase domain in place of the YTY motif found in other class 2 DYRKs; and the activation loop of the molecule begins with DLG rather than the more typical DFG motif.

**Figure 3:**
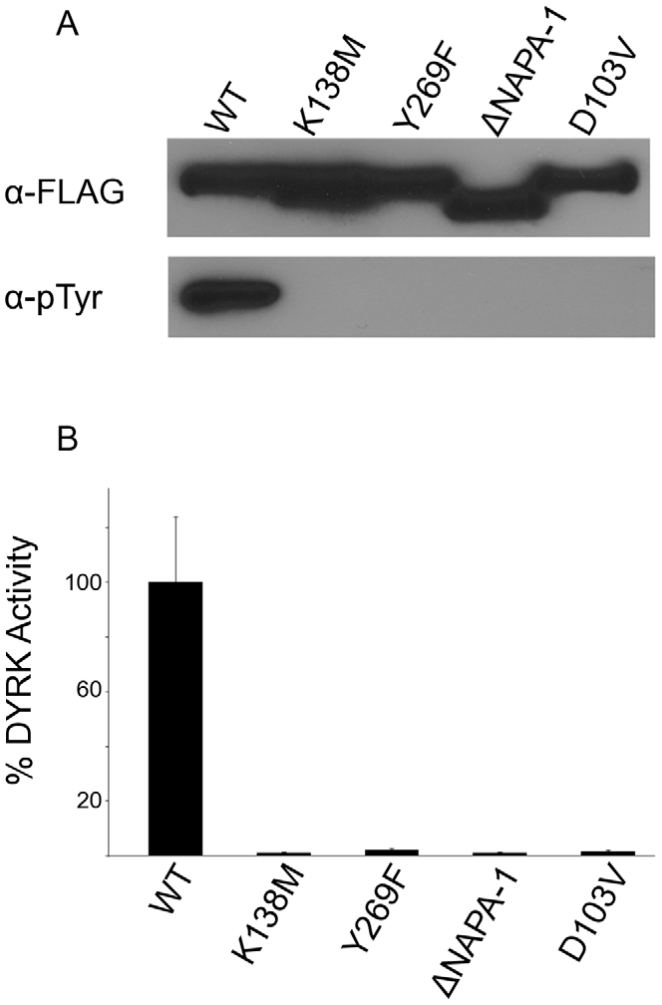
NAPA-1 and NAPA-2 are essential for TbDYRK2 activation loop phosphorylation. WT and mutant forms of TbDYRK2 were expressed in Sf9 cells and immunoprecipitated from cell extracts with anti-FLAG antibody. TbDYRK2 proteins analyzed include full length (WT), kinase inactive (K138M), activation loop tyrosine to phenylalanine (Y269F), NAPA-1-deletion lacking residues 53–76 (ΔNAPA-1), and point mutation of an invariant NAPA-2 residue (D103V). (A) Immunoprecipitates were subjected to SDS/PAGE and immunoblot analysis. Levels of TbDYRK2 proteins were detected with anti-FLAG antibody (α-FLAG), and activation loop phosphorylation was monitored with anti-phosphotyrosine antibody (α-pTyr). Experiments were performed at least three times. (B) Immunocomplexes of these same TbDYRK2 proteins were prepared in triplicate as above, and the kinase activity was measured using Woodtide as substrate as described in Materials and Methods. The results are presented as the means ± SD (n = 3).

For an initial characterization of TbDYRK2, we compared levels of tyrosine phosphorylation and the kinase activity of the WT molecule to a kinase-inactive form of the protein (K138M). We also compared the WT protein to an activation loop mutant (Y269F) where we modified the analogous tyrosine residue shown to be phosphorylated in other DYRKs. To monitor expression of WT and mutant proteins, a Flag-epitope was fused to the N-terminus and the modified proteins were expressed using recombinant baculovirus-infected Sf9 cells. When approximately equal levels of the proteins were compared, WT-TbDYRK2 was found to be tyrosine phosphorylated whereas the kinase-inactive and activation loop mutants displayed little if any tyrosine phosphorylation (Fig. 3A).

Next we compared the ability of the WT and mutant proteins to phosphorylate the DYRK substrate, Woodtide. For this assay, TbDYRK2 proteins were immunoprecipitated and the kinase activity of the immunocomplexes determined by incubation with the Woodtide peptide. The results paralleled the levels of tyrosine phosphorylation, the WT molecule was significantly more active than either the kinase-inactive or activation loop mutant (Fig. 3B). The activation loop tyrosine of TbDYRK2 has been shown to be phosphorylated in vivo in two independent studies [24], [46]. Based on these observations and the evidence presented here, we conclude TbDYRK2 behaves similarly to *Drosophila* and mammalian class 2 DYRKs with regard to activation loop tyrosine autophosphorylation and the importance of this phosphorylation event for full kinase activity.

### TbDYRK2 requires the NAPA-1 and 2 regions for tyrosine autophosphorylation

We recently demonstrated that the NAPA-1 and 2 regions were essential for tyrosine autophosphorylation and the subsequent kinase activity of *Drosophila* DYRK2 (DmDYRK2) [39]. To determine if the NAPA region function is conserved and is required for TbDYRK2 tyrosine autophosphorylation, we deleted the NAPA-1 region (residues 53–76), or modified the invariant NAPA-2 residue, Asp^103^ (see Fig. 2B for positional information of mutations), and expressed the proteins in Sf9 cells. These NAPA-1 and NAPA-2 mutations effectively abolished TbDYRK2 tyrosine autophosphorylation (Fig. 3A). In addition, the kinase activity of both NAPA mutants was also significantly impaired (Fig. 3B). These results provide strong evidence that the NAPA region has a critical and conserved role in activation loop autophosphorylation from Trypanosomes to Drosophila.

Normally, the NAPA-1 region functions in *cis* and removal of the first 167 N-terminal amino acids (which includes the NAPA-1 region) from DmDYRK2 (ΔN2-DmDYRK2) essentially eliminated activation loop tyrosine autophosphorylation. However, autophosphorylation could be rescued in *trans* by co-expression of the isolated N-terminus of the same DmDYRK2 molecule (residues 1–198) [39]. We used this trans-complementation assay to determine if the N-terminus of TbDYRK2, or of human DYRK2 (HsDYRK2), shared sufficient functional conservation to rescue autophosphorylation by the *Drosophila* ΔN2-DmDYRK2 protein.

**Figure 4:**
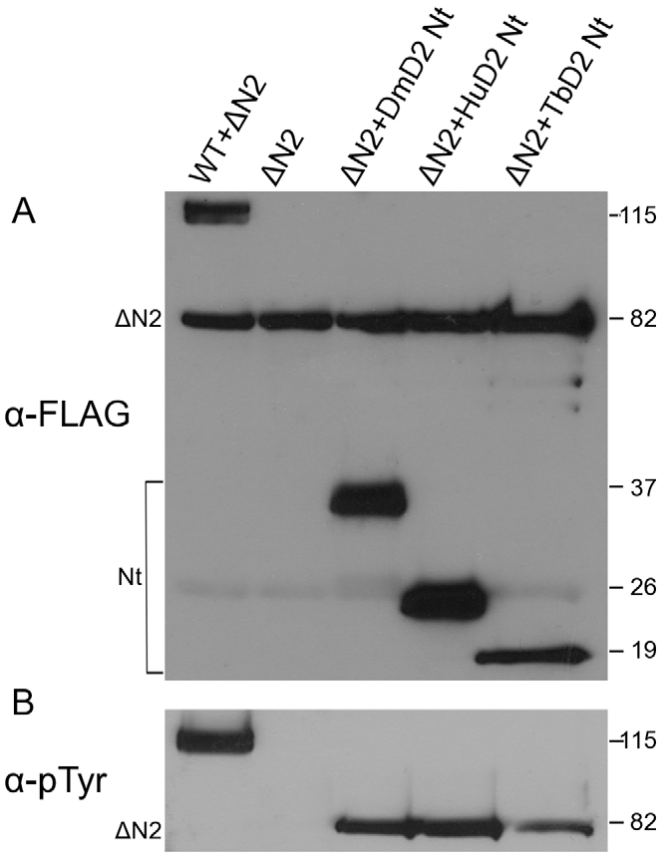
The NAPA-1 region is functionally conserved across species. The activation loop phosphorylation-defective mutant ΔN2-DmDYRK2 (ΔN2) was expressed alone in Sf9 cells; or it was coexpressed with full-length WT DmDYRK2 (WT), or with the entire non-catalytic N-terminus of either *Drosophila* DmDYRK2 (DmD2 Nt), human DYRK2 (HsD2 Nt), or *T. brucei* TbDYRK2 (TbDYRK2 Nt), as indicated. (A) Proteins were immunoprecipitated from Sf9 cell lysates with anti-FLAG antibody, fractionated by SDS/PAGE, and analyzed on immunoblots. Protein levels were monitored by probing with anti-FLAG antibody (α-FLAG). (B) WT and ΔN2-DmDYRK2 (proteins containing the kinase domain) were immunoprecipitated from cell lysates with an antibody against the C-terminus of DmDYRK2, fractionated by SDS/PAGE, and analyzed on immunoblots. The ability of the full-length DmDYRK2, and N-terminal DYRK2 proteins from *Drosophila*, human and *Trypanosoma* to complement tyrosine activation loop phosphorylation by ΔN2-DmDYRK2 in *trans* was assessed by probing blots with anti-pTyr antibody (α-pTyr). Results are representative of three different experiments.

The N-terminus of DmDYRK2 used in *trans*-complementation studies extends to the first residue of the kinase domain. We cloned FLAG-epitope tagged versions of the analogous region from TbDYRK2 (residues 1–109) and from HsDYRK2 (residues 1–149). We then expressed the activation loop phosphorylation-defective ΔN2-DmDYRK2 protein alone or co-expressed it with the N-terminus of DmDYRK2, TbDYRK2 or HsDYRK2 in Sf9 cells. Protein levels were normalized (Fig. 4A) and the extent of ΔN2-DmDYRK2 tyrosine phosphorylation determined (Fig. 4B). Tyrosine autophosphorylation by ΔN2-DmDYRK2 was severely compromised; and co-expression of the isolated N-terminus of DmDYRK2 rescued this defect in *trans* (Fig. 4B). Co-expression of the N-terminus of either TbDYRK2 or HsDYRK2 also efficiently rescued tyrosine autophosphorylation by ΔN2-DmDYRK2 (Fig. 4B).

We also created an analogous mutation in TbDYRK2 (ΔN2-TbDYRK2; see Fig. 2B for positional information), to determine if this mutant protein was compromised in activation loop phosphorylation, and if so if it was possible to rescue the defect in *trans*. Similar to the *Drosophila* counterpart, ΔN2-TbDYRK2 was not tyrosine autophosphorylated when expressed alone (Fig. S4). However, in contrast to the *Drosophila* protein, we were unable to complement activation loop phosphorylation in *trans* by co-expressing the N-terminus of TbDYRK2 (Fig. S4). We found that co-expression experiments with *Trypanosoma* were more difficult than those with the *Drosophila* proteins, and in order to obtain adequate amounts of ΔN2-TbDYRK2 for immunoblot analysis, immunoprecipitation reactions needed to be extended for significantly longer periods of time. While we believe the preponderance of evidence supports the functional conservation of the NAPA regions across eukaryotic supergroups, the clear difference in the behavior of the *Drosophila* and *Trypanosoma* ΔN2-proteins demonstrate that caution and further work are needed before this statement can be given with certainty.

### NAPA-1 is necessary and sufficient for trans-complementation

**Figure 5:**
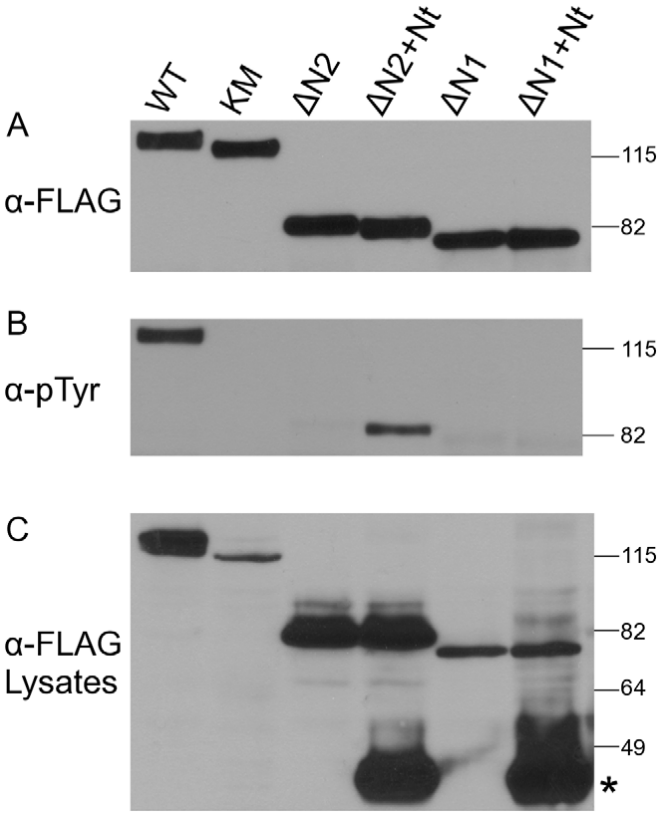
Activation loop phosphorylation by ΔN1-DmDYRK2 cannot be rescued in *trans*. The N-terminus (Nt) of DmDYRK2 was assessed for its ability to complement activation loop phosphorylation by ΔN1-DmDYRK2 (ΔN1) and ΔN2-DmDYRK2 (ΔN2). (A) ΔN1-DmDYRK2 and ΔN2-DmDYRK2 were expressed alone in Sf9 cells or they were co-expressed with Nt-DmDYRK2 (Nt), as indicated. WT- and kinase-inactive K227M-DmDYRK2 proteins were included as controls. (A and B) DmDYRK2 proteins containing the kinase domain were immunoprecipitated from cell lysates with anti-DmDYRK2 antibody, fractionated by SDS/PAGE and analyzed on immunoblots. (A) Levels of proteins were compared by probing blots with anti-FLAG antibody (α-FLAG) and (B) levels of activation loop autophosphorylation were assessed with anti-pTyr antibody (α-pTyr). (C) To confirm that all proteins were expressed, Sf9 cell lysates were fractionated by SDS/PAGE and the levels of the DmDYRK2 proteins determined by probing with anti-FLAG antibody (α-FLAG Lysates). The experiments shown were performed at least three separate times.

We also took this opportunity to explore the *trans*-activation reaction in greater detail. Previously we described two DmDYRK2 N-terminal deletion mutants that were defective in activation loop phosphorylation; ΔN2-DmDYRK2 (missing residues 1–167 described above), and a more extensive deletion ΔN1-DmDYRK2 missing residues 1–189 [39]. As shown (Figs. 4 and 5), activation loop phosphorylation by ΔN2-DmDYRK2 can be rescued in *trans* by co-expressing the isolated N-terminus of DmDYRK2. In contrast, co-expression of Nt-DmDYRK2 did not rescue tyrosine autophosphorylation by ΔN1-DmDYRK2 (Fig. 5).

**Figure 6:**
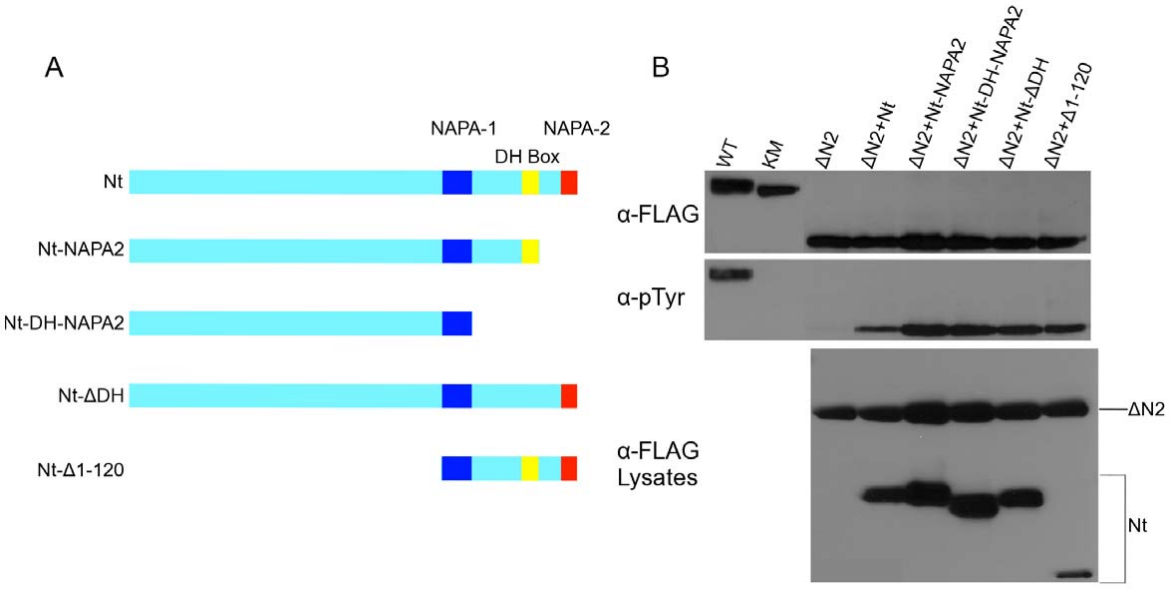
NAPA-1 is the critical region for *trans*-complementation. The N-terminal DmDYRK2 molecules shown schematically in (A) were assessed for their ability to complement activation loop phosphorylation by ΔN2-DmDYRK2. All proteins contain a FLAG epitope. The presence and location of the conserved NAPA-1, NAPA-2, and DH box regions within the truncated proteins are indicated. (B) ΔN2-DmDYRK2 was expressed alone in Sf9 cells or it was co-expressed with N-terminal truncated proteins as indicated. WT- and kinase-inactive K227M-DmDYRK2 proteins were included as controls. In the top two panels of B, DmDYRK2 proteins containing the kinase domain were immunoprecipitated from cell lysates with anti-DmDYRK2 antibody, fractionated by SDS/PAGE and analyzed on immunoblots. Levels of proteins were compared by probing blots with anti-FLAG antibody (α-FLAG) and levels of activation loop phosphorylation were assessed with anti-pTyr antibody (α-pTyr). To confirm that all proteins were expressed, Sf9 cell lysates were fractionated by SDS/PAGE and the levels of the DmDYRK2 proteins determined by probing with anti-FLAG antibody (α-FLAG Lysates). The experiments shown were performed at least three separate times.

For *trans*-complementation assays, we have used the entire non-catalytic region of DmDYRK2 (Nt-DmDYRK2) to rescue ΔN2-DmDYRK2 activation loop phosphorylation. Nt-DmDYRK2 contains 120 amino acids followed by the NAPA-1, DH-box, and NAPA-2 regions [39]. To further define the minimal portion of the N-terminus sufficient for *trans*-complementation, we generated four additional constructs (shown schematically in Fig. 6A): The Nt-Δ1–120 construct removes 120 amino acids prior to NAPA-1; Nt-ΔNAPA2 removes the NAPA-2 region; Nt-ΔDH replaces the DH-box with alanine residues; and Nt-ΔDH-ΔNAPA2 deletes both the NAPA-2 and DH-box regions. We then tested the ability of these different constructs to rescue activation loop phosphorylation by ΔN2-DmDYRK2 by co-expressing the proteins in Sf9 cells and monitoring ΔN2-DmDYRK2 for tyrosine phosphorylation. All four of the new truncated mutants efficiently complemented ΔN2-DmDYRK2 activation loop phosphorylation in *trans* (Fig. 6B).

Taken together, these results indicate that the NAPA-1 region appears to be both necessary and sufficient for trans-complementation of ΔN2-DmDYRK2. In contrast, these results indicate that the DH-box and NAPA-2 regions may be required in cis for efficient activation loop tyrosine autophosphorylation.

## Discussion

In this study we show that the DYRK-family of protein kinases has deep evolutionary roots with family members present in all four eukaryotic supergroups for which genome sequencing data is available (Fig. 1, Fig. S2, Table S1). This analysis provides strong support that DYRK family members were in existence in the primordial or root eukaryote which gave rise to all known extant eukaryotic life forms. The identified DYRKs from 21 genomes show a clear clustering into 5 subfamilies as noted by others [15]: class 1, class 2, Yaks, HIPKs and Prp4s (Fig. 1, Fig. S2, Table S1). Of these, the HIPKs are clearly more recent as they are found only in metazoans of the Unikonts supergroup. Class 1 DYRKs were observed in only two of the supergroups, Chromalveolates and Unikonts, suggesting that this subfamily is also of a more recent divergence. The presence of Yaks and Prp4s in three of the four supergroups (absent from Excavates), indicates that the origins of these subfamilies are ancient. However, only Class 2 DYRKs were found in all four supergroups (Fig. 1, Fig. S2, Table S1). Our analysis of the DYRKs into five subfamilies was based on a comparison of kinase catalytic domains. Therefore, the presence of the NAPA and DH-box regions were found independent of the original clustering and suggest that this region can be identified by virtue of the sequence identity of the associated kinase catalytic domain, that the NAPA regions are diagnostic for class 2 DYRKs, and that they were present in the primordial or root DYRK.

The presence of DYRK-like proteins has been noted in trypanosomes [46], [48]. These proteins were identified using a more limited dataset of known DYRK family members. In the present more rigorous analysis, only one class 2 DYRK from excavates consistently clustered with known DYRKs. The additional DYRK-like proteins present in trypanosomatids may represent previously unknown DYRK subfamilies but such a conclusion will require a broader phylogenomic survey of excavates.

Previously, we identified regions (termed NAPA-1 and 2) in the non-catalytic N-terminus of *Drosophila* DmDYRK2 essential for activation loop tyrosine autophosphorylation [39]. The sequence of the NAPA-region is conserved; however, as these regions have been functionally evaluated only in DmDYRK2, we wished to determine if the NAPA-regions are likely to play a similar autophosphorylation-accessory role in other class 2 DYRKs. Due to its evolutionary distance from *Drosophila* as well as its importance in human health, we choose to analyze the single class 2 DYRK encoded by the trypanosomatid, *T. brucei* (TbDYRK2).

*T. brucei* is the causative agent of African trypanosomiasis or Sleeping Sickness and is one of three trypanosomatids that cause major human disease [47]. The other disease-causing trypanosomatids, *T. cruzi* (Chagas Disease) and *Leishmania major* (leishmaniasis) also encode a single class 2 DYRK (TcDYRK2 and LmDYRK2, respectively). The non-catalytic N-terminus of all three trypanosomatid DYRKs are relatively short, ranging in size from 82–112 amino acids. When aligned, homology between these three DYRKs begins with the first residue of the NAPA-1 region and ends with the final residue of the kinase domain (Fig. S5). The C-terminal regions of TbDYRK2 and TcDYRK2 are also small comprising 47 and 49 amino acids, respectively. In contrast, LmDYRK2 has 575 C-terminal residues (Fig. S5). As with other class 2 DYRKs, the C-termini of the three trypanosomatid DYRKs share little or no homology with each other or with other proteins.

As noted, TbDYRK2 contains an atypical DLG triplet at the beginning of the activation loop, a sequence shared with *L. major* LmDYRK2, whereas TcDYRK2 contains the more conventional activation loop DFG motif. The activation loop of TbDYRK2 also contains an unusual FTY sequence rather than the YTY motif found in other class 2 DYRKs (including TcDYRK2 and LmDYRK2). However, for all DYRKs thus far analyzed, it is only the second tyrosine in the YTY motif that is phosphorylated and essential for full serine/threonine kinase activity [3], [6], [9], [12], [14], [16], [24], [39]; and the analogous tyrosine in TbDYRK2 was shown to be phosphorylated *in vivo* in two separate studies that analyzed the phospho-proteome of *T. brucei* [46], [48]. It is also worth noting that the threonine in the activation loop YTY motif was absolutely conserved in all class 2 DYRKs. This residue was shown to be phosphorylated by MAP3K10 leading to a decrease in human DYRK2 catalytic activity [29]. This study and absolute conservation suggest that this residue may play an important role in regulating kinase activity.

In this study we have provided evidence that WT-TbDYRK2 is tyrosine phosphorylated (Fig. 3A). We also demonstrate that the NAPA-1 and 2 regions were essential for efficient activation loop phosphorylation of TbDYRK2, as deletion of NAPA-1 or altering a single NAPA-2 residue in TbDYRK2 essentially eliminated tyrosine autophosphorylation (Fig. 3A). The loss of tyrosine phosphorylation also resulted in a similar dramatic reduction in the serine/threonine kinase activity of the protein (Fig. 3B). We conclude that TbDYRK2 is phosphorylated on the same conserved activation loop tyrosine as described for other DYRKs [3], [6], [9], [12], this phosphorylation event is necessary for full catalytic activity of the enzyme, and that the NAPA-1 and 2 regions play an essential conserved role in this process (Figs. 3 and 4). Finally, the ability of the N-terminal region of TbDYRK2 or human DYRK2 to rescue activation loop phosphorylation by the *Drosophila* ΔN2-DmDYRK2 mutant protein (Fig. 4) demonstrates the functional conservation of this region across species and ∼850 million years of independent evolution [49].

## Supporting Information

Figure S1 Nucleotide sequence and conceptual translation of TbDYRK2.
(DOC)

Figure S2 Phylogenetic tree of the DYRKs. DYRK family members identified in our search of 21 sequenced genomes clustered into five separate subfamilies: class 1, class 2, HIPK, PRP4, and Yak.
(TIF)

Figure S3 Multiple protein alignment of class 2 DYRKs.
(TIF)

Figure S4 WT and mutant forms of TbDYRK2 were expressed in Sf9 cells and immunoprecipitated with anti-FLAG antibody. TbDYRK2 proteins analyzed include full length (WT), kinase inactive (K138M), activation loop tyrosine to phenylalanine (Y269F), N-terminal -deletion lacking residues 1–78 (ΔN2), and the entire non-catalytic N-terminus (Nt) of the molecule. ΔN2 was expressed alone in Sf9 cells; or it was coexpressed with Nt as indicated. Immunoprecipitates were subjected to SDS/PAGE and immunoblot analysis. Levels of TbDYRK2 proteins were detected with anti-FLAG antibody (α-FLAG), and activation loop phosphorylation was monitored with anti-phosphotyrosine antibody (α-pTyr). Large (*) and small (**) IgG background bands are indicated. Experiments were performed at least three times.
(TIF)

Figure S5 CLUSTAL W (1.83) multiple sequence alignment of class 2 DYRKs from the trypanosomatids *L. major*, *T. brucei*, and *T. cruzi*. Symbols * invariant, : conservative substitutions, and . semi-conserved substitutions.
(DOC)

Table S1 Identifiers of individual DYRK subfamily members categorized in each eukaryotic supergroup.
(DOCX)
