# Supplementary material for: Deep Evolutionary Conservation of an Intramolecular Protein Kinase Activation Mechanism
Source: PLoS One. 2012 Jan 3;7(1):e29702. doi: 10.1371/journal.pone.0029702 (PMC3250476; doi:10.1371/journal.pone.0029702)
Supplement: Figure S1 — Nucleotide sequence and conceptual translation of TbDYRK2. (DOC) [file pone.0029702.s001.doc]

**M D V S R R L A G E N H D F H R S T Q V P A L C T** atggatgtctctcgcaggttggcgggtgaaaaccatgacttccacaggagcacgcaggttcctgcgctctgtacg

**k p q g g k g m r l n v l h e g a v p a n d f h s**

aaaccgcaagggggcaaggggatgcggctaaatgtgttgcacgaaggtgcagtccccgctaatgactttcattcc

**a h l t p y e m d e i k g y s e v y y v g q n c d**

gctcatcttactccgtacgagatggacgagataaagggctactcggaagtatattacgttggccaaaattgtgac

**r k v q a p v e g g h n k g y d d e r g d y l i r**

cgcaaggttcaggcaccggtggaaggaggccataacaaggggtatgatgatgaaagaggggactacctcatcaga

**l r d h i a y r y e v l s t l g s g s f g q v v k**

ctacgtgaccacatcgcataccgctacgaggtcttaagcacactggggagtggttccttcggacaggtggttaag

**a v d h c k n c t v a l k i i r n r k r f t a q a**

gcagttgatcactgcaagaactgtactgtagcgctaaagattatccgcaacaggaaacgcttcaccgcacaagca

**k i e v q i l s h l k k g d p s g i y g i v q m i**

aagattgaggtgcagattttgagtcatttgaagaaaggtgacccctcagggatctacggaatagtccaaatgatt

**d n f t f r s h v c i t y e l l g c n l y t y l k**

gataatttcacctttcgttcccatgtctgcattacatacgagctgttaggatgtaacttgtacacttacttaaag

**q r r f k p l p l d i v r k i g a g v l v s l s y**

caacgcaggttcaagccattgccgttagacatcgttcgcaagattggggctggtgttctcgtgtctctttcctac

**m w r e n i i h c d l k p e n i l l r s p n d t a**

atgtggcgagagaacattattcattgtgatctgaagccggaaaacatactcctcaggtcacccaatgacacggca

**v k v i d l g s s c f e n a r l f t y i q s r f y**

gtgaaagtgattgacttgggctcctcttgctttgaaaacgcccgtttgttcacctacatccaatcgcgcttctac

**r a p e v l l g c p y s r c i d l w s y g c v l c**

cgtgcacccgaggtacttctagggtgtccctactccaggtgcatagatctttggagttatggatgtgttctttgc

**e l a s g y p i f p g e s e q e q m a c i m e f l**

gagctcgcctccggctaccccattttcccaggtgaaagcgagcaggaacagatggcgtgcataatggagttttta

**g t p p r d f i l r s p r k h e f f e a s a n y s**

gggaccccgccgcgtgattttatacttcgttccccgcgcaaacatgagttttttgaagcaagcgcaaattattca

**p k l v p n s k l k i r f p g t k n i a a f l g l**

cccaaattagttccaaacagcaaacttaaaatacgctttcctggaaccaagaatattgcggcgttccttggactg

**p e g d p f v s f v k l f l e w v p d s r a t p r**

ccggagggagatccatttgtcagtttcgttaagctgttccttgagtgggttccggactcgcgtgccactcctcgg

**r a m k h p w i a d e v n e l l s k q k r n t a a**

cgggcgatgaagcacccgtggatcgctgatgaggtcaatgaattattaagtaagcaaaagcggaacacagcggcc

**t g e d d s e s k f h k a l p r l p k i g k r g t**

actggcgaagatgatagtgagtctaagttccacaaagcccttccgcgcttgcctaagataggcaaacgtgggacc

**d r c g c ***

gacagatgcgggtgctag
