# Supplementary figures and images for: Deep Evolutionary Conservation of an Intramolecular Protein Kinase Activation Mechanism
Source: PLoS One. 2012 Jan 3;7(1):e29702. doi: 10.1371/journal.pone.0029702 (PMC3250476; doi:10.1371/journal.pone.0029702)

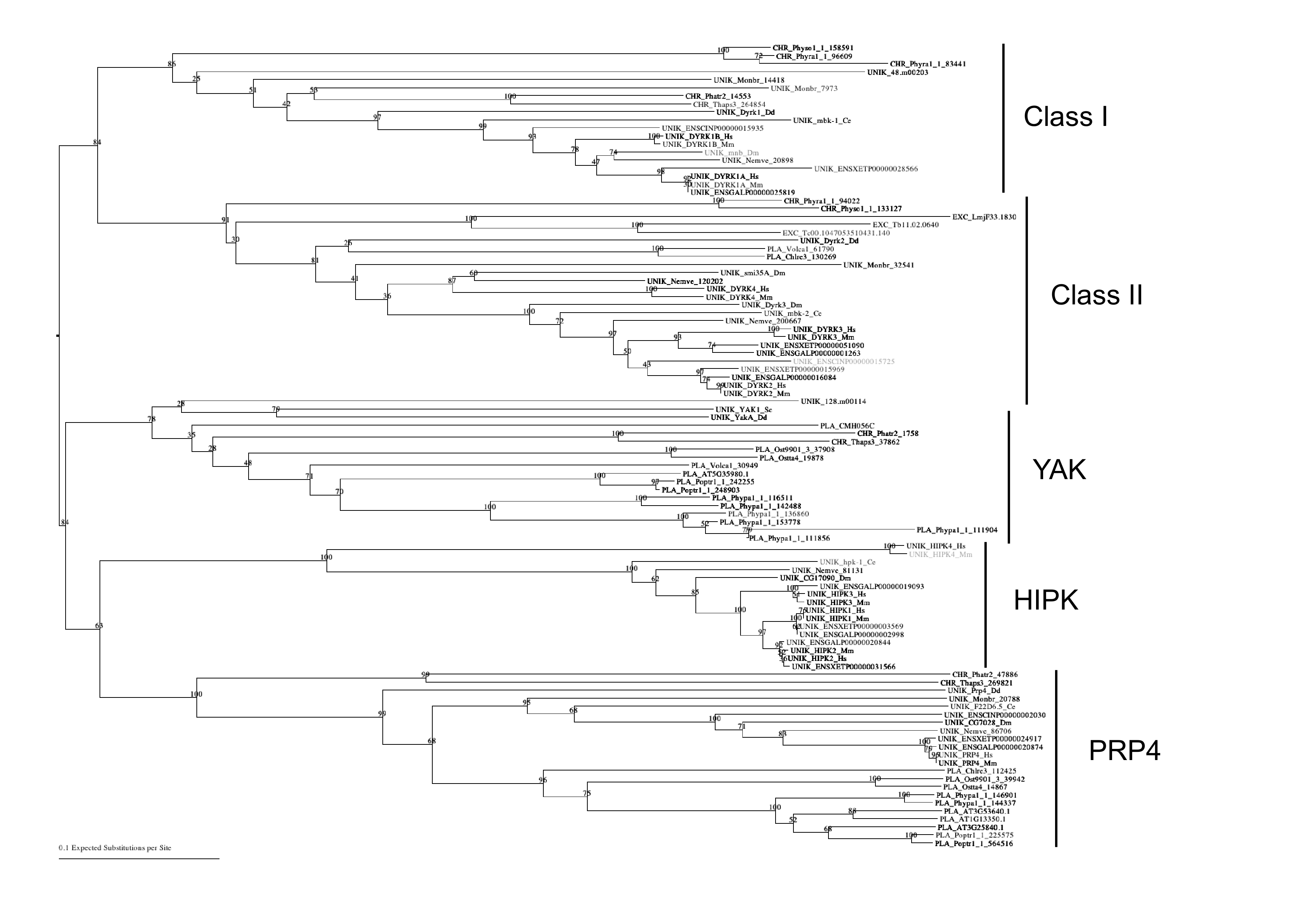

Supplement: Figure S2 — Phylogenetic tree of the DYRKs. DYRK family members identified in our search of 21 sequenced genomes clustered into five separate subfamilies: class 1, class 2, HIPK, PRP4, and Yak. (TIF) [file pone.0029702.s002.tif]

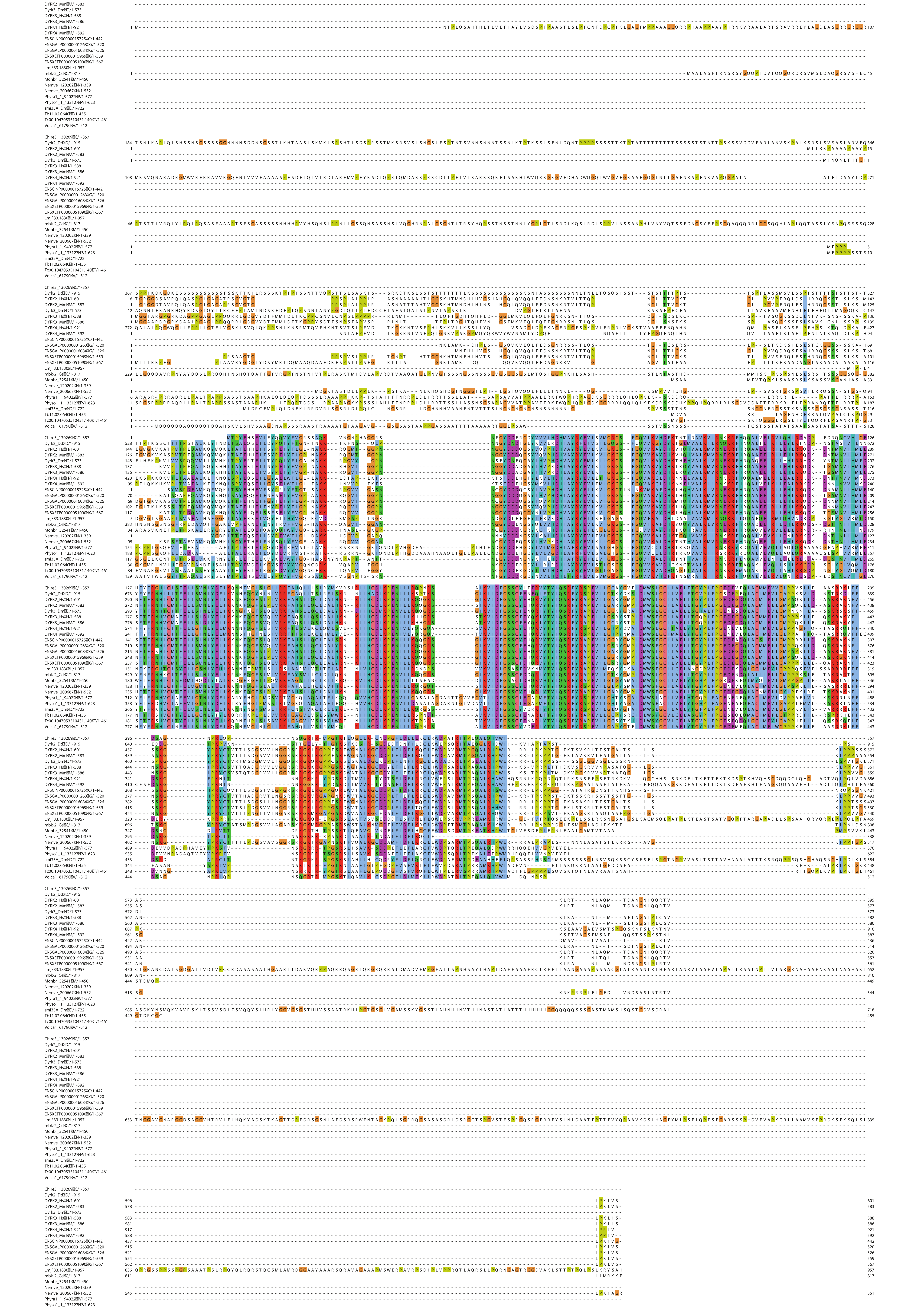

Supplement: Figure S3 — Multiple protein alignment of class 2 DYRKs. (TIF) [file pone.0029702.s003.tif]

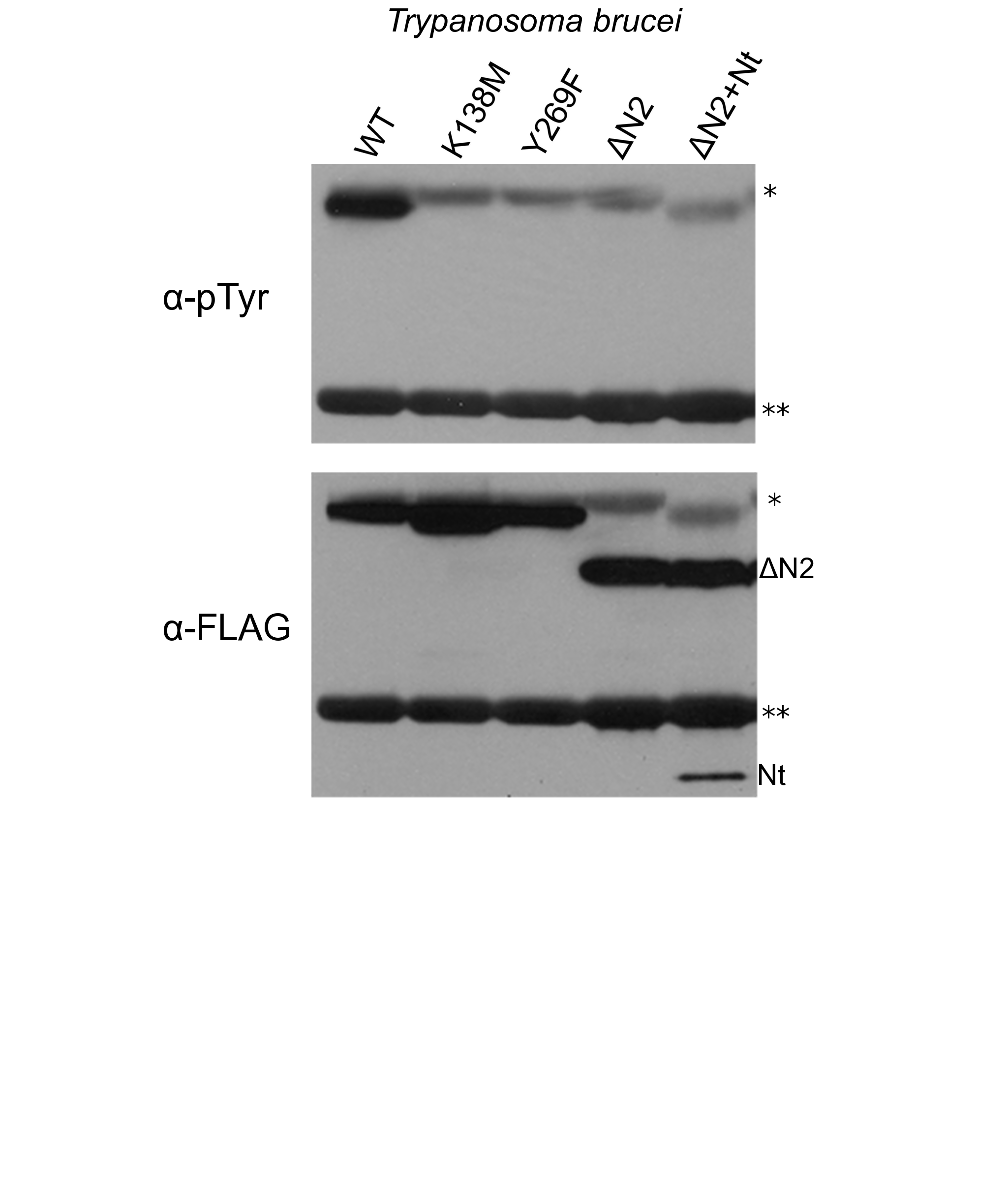

Supplement: Figure S4 — WT and mutant forms of TbDYRK2 were expressed in Sf9 cells and immunoprecipitated with anti-FLAG antibody. TbDYRK2 proteins analyzed include full length (WT), kinase inactive (K138M), activation loop tyrosine to phenylalanine (Y269F), N-terminal -deletion lacking residues 1–78 (ΔN2), and the entire non-catalytic N-terminus (Nt) of the molecule. ΔN2 was expressed alone in Sf9 cells; or it was coexpressed with Nt as indicated. Immunoprecipitates were subjected to SDS/PAGE and immunoblot analysis. Levels of TbDYRK2 proteins were detected with anti-FLAG antibody (α-FLAG), and activation loop phosphorylation was monitored with anti-phosphotyrosine antibody (α-pTyr). Large (*) and small (**) IgG background bands are indicated. Experiments were performed at least three times. (TIF) [file pone.0029702.s004.tif]
