# Supplementary material for: Deep Evolutionary Conservation of an Intramolecular Protein Kinase Activation Mechanism
Source: PLoS One. 2012 Jan 3;7(1):e29702. doi: 10.1371/journal.pone.0029702 (PMC3250476; doi:10.1371/journal.pone.0029702)
Supplement: Figure S5 — CLUSTAL W (1.83) multiple sequence alignment of class 2 DYRKs from the trypanosomatids L. major, T. brucei, and T. cruzi. Symbols * invariant, : conservative substitutions, and . semi-conserved substitutions. (DOC) [file pone.0029702.s005.doc]

Lmajor -----------------------------MHPEDGVGTSGAAPISVSEALHSFGGCLTAY

Tbrucei ----MDVSRRLAGENHDFRRSTQVPALCTKPQGGKGMRLNVLHEGAVPANDFHSAHLTPY

Tcruzi MYGTSKIQGGGLRGSLHYCTQQRFLPSANRTPGFVNARGKVTASKAATSSEYHAAYLTEY

. . : . ... ** *

Lmajor ERKEIVQYETIHYVGQRCVDKMGSPTNG-RNDGYDTEEGEYIFRVKDHIAYRYEILKELG

Tbrucei EMDEIKGYSEVYYVGQNCDRKVQAPVEGGHNKGYDDERGDYLIRLRDHIAYRYEVLSTLG

Tcruzi EKIEIRGYKDVYYVGQNCERKIQAPVEGGYNCGYDDERGDYTIMLHDHIAYRYEVLGTLG

* ** *. ::****.* *: :*.:* * *** *.*:* : ::********:* **

Lmajor SGAFGQVFKAIDHLDSSIVAVKMIRNQRKILQQAEQEIRMLQHVNDRDPKGLYGIVRMTD

Tbrucei SGSFGQVVKAVDHCKNCTVALKIIRNRKRFTAQAKIEVQILSHLKKGDPSGIYGIVQMID

Tcruzi SGSFGQVVKVADHQNGTTVALKIIRNKKRFMSQAKIEVQILSHLRRGDPNGIYGIVQMLD

**:****.*. ** .. **:*:***:::: **: *:::*.*:. **.*:****:* *

Lmajor NFKFRGHTCISYELLGSNLYEHLKANNFFPMTLSLIRSIAARMLVTLTFLARENIVHCDL

Tbrucei NFTFRSHVCITYELLGCNLYTYLKQRRFKPLPLDVVRKIGAGVLVSLSYMWRENIIHCDL

Tcruzi SFTFRSHVCITYELLSINLYEHLKQRNFHPLSLSAVRKIGAGVLVSLSYIWRENIIHCDL

.*.**.*.**:****. *** :** ..* *:.*. :*.*.* :**:*::: ****:****

Lmajor KPENILLRDNDPSVVKVIDLGSASFDVKNMYTYIQSRFYRAPEVIMEQKYDKAIDWWSFG

Tbrucei KPENILLRSPNDTAVKVIDLGSSCFENARLFTYIQSRFYRAPEVLLGCPYSRCIDLWSYG

Tcruzi KPENILLKTPDRATVKVIDFGSSCFENARIYTYIQSRFYRAPEVILGCSYTKHIDLWSYG

*******: : :.*****:**:.*: .::*************:: * : ** **:*

Lmajor CILCELANGDPVFPGEDEKDQLGCIMEYLGPPPQSFVEISSARRRREFFDERYKPRPRTT

Tbrucei CVLCELASGYPIFPGESEQEQMACIMEFLGTPPRDFILRSPRKHEFFEASANYSPKLVPN

Tcruzi CVLCELATGVPIFPGESEQDQLGCIMEYLGAPPHELILQSSRKQELFDVNNGYAPKLVPN

*:*****.* *:****.*::*:.****:**.**:.:: *. ::. . * *: ..

Lmajor CKGKQREPGSRSLAKFVSVSEDDDFLSFVRLFLQWEPSKRVPPREAMKHRWICGEFVFPT

Tbrucei SKLKIRFPGTKNIAAFLGLPEGDPFVSFVKLFLEWVPDSRATPRRAMKHPWIADEVN---

Tcruzi SRRKIRYPGTRSLAAFLGLPQDDGFVSFVRQFLCWIPEERVSPRRAMRHPWIADIFEGPP

.: * * **::.:* *:.:.:.* *:***: ** * *..*..**.**:* **.. .

Lmajor QSEEKPELSSLRKSNGSAIGSLKACMSQEPATPLKTEASTSATVGQPFTARGAPADLLSP

Tbrucei ---ELLSKQKRNTAATGED-----------------------------------------

Tcruzi PPLSQVSKTQTNLAVRAAI-----------------------------------------

. . . . : .

Lmajor SAAHQRVQRPEPLPQLPTACTGRANCDALSGDGAILVDTVPCCRDASASAATHGAARLTD

Tbrucei DSESKFHKALPRLPKIGKRGTDRCGC----------------------------------

Tcruzi SNAHRITGQPLKVPHLPKIGEH--------------------------------------

. : :*:: .

Lmajor AKVQRPPAQRRQSGRLQRGRQRRSTDMADVEMPGEAITSPNHSAYLHAPLDAEESSAERC

Tbrucei ------------------------------------------------------------

Tcruzi ------------------------------------------------------------

Lmajor TREFIIAANGASSPSSSACGTATRASNTRLHEARLANRVLSSEVLSPAILRSSTNPIIVT

Tbrucei ------------------------------------------------------------

Tcruzi ------------------------------------------------------------

Lmajor SRGRNAHSAENKASTNASHSKITNGGAVGNARGGDSAGGVHTRVLELHQKYADSKTKAGT

Tbrucei ------------------------------------------------------------

Tcruzi ------------------------------------------------------------

Lmajor TDPFDRSGSNIAFDSRSRWFNTAGKPQLSGRRQGSASASDRLDSRGCTSPGVSTESPRGQ

Tbrucei ------------------------------------------------------------

Tcruzi ------------------------------------------------------------

Lmajor SRGERREYSINLDAATFPTTEVYQPAAVKDSLHAGEYMLPSELQPFSEGARSSSPHDVEV

Tbrucei ------------------------------------------------------------

Tcruzi ------------------------------------------------------------

Lmajor APKCRLLAAMVSEPRDKSEKSQLSLQPRGSSPPSSPGPSAAATPSLRPQYQLRQRSTQCS

Tbrucei ------------------------------------------------------------

Tcruzi ------------------------------------------------------------

Lmajor MLAMRDGGAAYAAARSQRAVAGAAAPMSWERPAVRPSDIPLVPPRQTLAQRSLLPQRNGA

Tbrucei ------------------------------------------------------------

Tcruzi ------------------------------------------------------------

Lmajor GTRGGDVAKLSTTPTPQLPSLKRYSAH

Tbrucei ---------------------------

Tcruzi ---------------------------
