# Supplementary material for: Deep Evolutionary Conservation of an Intramolecular Protein Kinase Activation Mechanism
Source: PLoS One. 2012 Jan 3;7(1):e29702. doi: 10.1371/journal.pone.0029702 (PMC3250476; doi:10.1371/journal.pone.0029702)
Supplement: Table S1 — Identifiers of individual DYRK subfamily members categorized in each eukaryotic supergroup. (DOCX) [file pone.0029702.s006.docx]

|  | **DYRK classification** | | | | |
| --- | --- | --- | --- | --- | --- |
| **Taxonomy and species** | **Class I** | **Class II** | **YAK** | **HIPK** | **PRP4** |
| **Unikonts** | | | | | |
| *Caenorhabditis elegans* | mbk-1_Ce | mbk-2_Ce |  | hpk-1_Ce | Prp4_Dd |
| *Ciona intestinalis* | ENSCINP00000015935 | ENSCINP00000015725 |  |  | ENSCINP00000002030 |
| *Dictyostelium discoideum* | Dyrk1_Dd | Dyrk2_Dd | YakA_Dd |  | F22D6.5_Ce |
| *Drosophila melanogaster* | mnb_Dm | smi35A_Dm, Dyrk3_Dm |  | CG17090_Dm | CG7028_Dm |
| *Entamoeba histolytica* | 48.m00203 |  | 128.m00114 |  |  |
| *Gallus gallus* | ENSGALP00000025819 | ENSGALP00000001263, ENSGALP00000016084 |  | ENSGALP00000002998, ENSGALP00000020844, ENSGALP00000019093 | ENSGALP00000020874 |
| *Homo sapiens* | DYRK1A_Hs, DYRK1B_Hs | DYRK2_Hs, DYRK3_Hs, DYRK4_Hs |  | HIPK1_Hs, HIPK2_Hs, HIPK3_Hs, HIPK4_Hs | PRP4_Hs |
| *Monosiga brevicollis* | Monbr_14418, Monbr_7973 | Monbr_32541 |  |  | Monbr_20788 |
| *Mus musculus* | DYRK1A_Mm, DYRK1B_Mm | DYRK2_Mm, DYRK3_Mm, DYRK4_Mm |  | HIPK1_Mm, HIPK2_Mm, HIPK3_Mm, HIPK4_Mm | PRP4_Mm |
| *Nematostella vectensis* | Nemve_20898 | Nemve_120202, Nemve_200667 |  | Nemve_81131 | Nemve_86706 |
| *Saccharomyces cerevisiae* |  |  | YAK1_Sc |  |  |
| *Xenopus tropicalis* | ENSXETP00000028566 | ENSXETP00000051090, ENSXETP00000015969 |  | ENSXETP00000031566, ENSXETP00000003569 | ENSXETP00000024917 |
|  |  |  |  |  |  |
| **Chromalveolates** |  |  |  |  |  |
| *Phaeodactylum tricornutum* | Phatr2_14553 |  | Phatr2_1758 |  | Phatr2_47886 |
| *Phytophthora ramorum* | Phyra1_1_96609, Phyra1_1_83441 | Phyra1_1_94022 |  |  |  |
| *Phytophthora sojae* | Physo1_1_158591 | Physo1_1_133127 |  |  |  |
| *Thalassiosira pseudonana* | Thaps3_264854 |  | Thaps3_37862 |  | Thaps3_269821 |
| **Excavates** | | | | | |
| *Leishmania major* |  | LmjF33.1830 |  |  |  |
| *Trypanosoma brucei* |  | Tb11.02.0640 |  |  |  |
| *Trypanosoma cruzi* |  | Tc00.1047053510431.140 |  |  |  |
| **Plants** | | | | | |
| *Arabidopsis thaliana* |  |  | AT5G35980.1 |  | AT3G53640.1, AT1G13350.1, AT3G25840.1 |
| *Chlamydomonas reinhardtii* |  | Chlre3_130269 |  |  | Chlre3_112425 |
| *Cyanidioschyzon merolae* |  |  | CMH056C |  |  |
| *Ostreococcus lucimarinus* |  |  | Ost9901_3_37908 |  | Ost9901_3_39942 |
| *Ostreococcus tauri* |  |  | Ostta4_19878 |  | Ostta4_14867 |
| *Physcomitrella patens* |  |  | Phypa1_1_116511, Phypa1_1_142488, Phypa1_1_136860, Phypa1_1_153778, Phypa1_1_111904, Phypa1_1_111856 |  | Phypa1_1_146901, Phypa1_1_144337 |
| *Populus trichocarpa* |  |  | Poptr1_1_242255, Poptr1_1_248903 |  | Poptr1_1_225575, Poptr1_1_564516 |
| *Volvox carteri* |  | Volca1_61790 | Volca1_30949 |  |  |
